# Rationale and design of the iPap trial: a randomized controlled trial of home-based HPV self-sampling for improving participation in cervical screening by never- and under-screened women in Australia

**DOI:** 10.1186/1471-2407-14-207

**Published:** 2014-03-19

**Authors:** Farhana Sultana, Dallas R English, Julie A Simpson, Julia ML Brotherton, Kelly Drennan, Robyn Mullins, Stella Heley, C David Wrede, Marion Saville, Dorota M Gertig

**Affiliations:** 1 Centre for Epidemiology and Biostatistics, Melbourne School of Population and Global Health, University of Melbourne, Melbourne, Australia; 2 Cancer Council Victoria, 615 St Kilda Road, Melbourne, Australia; 3 National HPV Vaccination Program Register, Victorian Cytology Service, PO Box 310, East Melbourne, Vic, 3002, Australia; 4 VCS Inc, 265 Faraday Street, Carlton, Vic, 3053, Australia; 5 Royal Women’s Hospital, Locked Bag 300, Cnr Flemington Road and Grattan Street, Parkville, Victoria, 3052, Australia; 6 VCS Pathology, 265 Faraday Street, Carlton, Vic, 3053, Australia; 7 Victorian Cervical Cytology Registry, PO Box 161, Carlton South, Vic, 3053, Australia

**Keywords:** HPV DNA testing, Home-based, Self-sample, Cervical screening, Participation

## Abstract

**Background:**

Organized screening based on Pap tests has substantially reduced deaths from cervical cancer in many countries, including Australia. However, the impact of the program depends upon the degree to which women participate. A new method of screening, testing for human papillomavirus (HPV) DNA to detect the virus that causes cervical cancer, has recently become available. Because women can collect their own samples for this test at home, it has the potential to overcome some of the barriers to Pap tests. The iPap trial will evaluate whether mailing an HPV self-sampling kit increases participation by never- and under-screened women within a cervical screening program.

**Methods/Design:**

The iPap trial is a parallel randomized controlled, open label, trial. Participants will be Victorian women age 30–69 years, for whom there is either no record on the Victorian Cervical Cytology Registry (VCCR) of a Pap test (never-screened) or the last recorded Pap test was between five to fifteen years ago (under-screened). Enrolment information from the Victorian Electoral Commission will be linked to the VCCR to determine the never-screened women. Variables that will be used for record linkage include full name, address and date of birth. Never- and under-screened women will be randomly allocated to either receive an invitation letter with an HPV self-sampling kit or a reminder letter to attend for a Pap test, which is standard practice for women overdue for a test in Victoria. All resources have been focus group tested. The primary outcome will be the proportion of women who participate, by returning an HPV self-sampling kit for women in the self-sampling arm, and notification of a Pap test result to the Registry for women in the Pap test arm at 3 and 6 months after mailout. The most important secondary outcome is the proportion of test-positive women who undergo further investigations at 6 and 12 months after mailout of results.

**Discussion:**

The iPap trial will provide strong evidence about whether HPV self-sampling could be used in Australia to improve participation in cervical screening for never-and under-screened women.

**Trial registration:**

ANZCTR Identifier: ACTRN12613001104741; UTN: U1111-1148-3885

## Background

Organized screening programs based on Pap tests have substantially reduced deaths from cervical cancer in resource rich settings [1], including Australia [2]. However, some women are missing out on the benefit because they do not have regular Pap tests [3]. About half (54%) of women diagnosed with invasive or micro-invasive cervical cancer in Victoria, Australia, have no known screening history and a further 25% were last screened more than 2.5 years before diagnosis [4]. Inclusion of these women into screening programs is crucial to further reducing cervical cancer incidence and mortality.

Numerous strategies have been used to improve participation in cervical screening, but most have had limited success, particularly in engaging ‘hard to reach’ groups. Common barriers include test-related issues such as pain, discomfort or embarrassment, or doctor-related issues including access, difficulty obtaining appointments or time constraints [5-9]. Reminder letters have been shown to be one of the most effective strategies at prompting women to re-attend [10]. In Victoria, a reminder letter is sent once a woman has not attended by 27 months since her last negative Pap test and another reminder is sent at 36 months if she has not responded to the first reminder. About 40% women had a subsequent Pap smear within three months of receiving the first reminder letter [4]. To increase participation in Victoria, a pilot study of 10,000 second reminder letters to women who did not respond to the first reminder was conducted in June 2011. Whilst the intervention was effective, the response rate decreased with increasing time since the last Pap test, ranging from 10% for women whose last Pap test was six years ago to 0.4% for women whose last Pap test was more than fifteen years ago [11].

Testing for DNA of the human papillomavirus (HPV), the virus that causes cervical cancer, has been evaluated as a primary screening test over the last decade [12-15]. Evidence from randomized trials suggests that HPV testing is more sensitive than Pap testing and has a better negative predictive value [16,17]. Furthermore, unlike a Pap test, women can collect their own samples for HPV testing. Self-collected samples have better sensitivity than Pap test and comparable sensitivity to those obtained by physicians [18-21].

### Rationale

Eight randomised controlled trials of HPV self-sampling evaluating whether it improves participation in screening have been reported from countries with organised screening programs (Table 1) [22-29]. The studies were restricted to non-attendees, although the eligibility criteria varied. Non-attendees in these studies referred to women who did not respond to an initial invitation or a 6 months reminder letter and who were overdue between three months and six years or more. All studies compared invitations to perform HPV self-sampling with invitations to attend for Pap tests, with HPV self-sampling kits mailed to women and returned by mail. Women in the comparison arm received a standard invitation letter or a reminder letter to attend for a Pap test. Six of the trials used Hybrid Capture II for their HPV DNA test [22-27], while one study used Abbott Real Time HPV test [29] and another study used GP5+/6+ PCR testing [28].

**Table 1:** Review of trials comparing participation in HPV self-sampling (SS) and reminder letter to attend for a Pap test

**Study**	**Area**	**Eligibility**	**Intervention**	**Comparison**	**Device**	**Test, HPV+**	**Participation**		**Follow-up**
							**HPV SS**	**Pap arm**	
Sancho-Garnier [29]	France	35-69 yrs; no Pap smear for ≥2 years; did not respond to first invitation	HPV SS kit*	Standard invitation	Dacron swab	Abbott real Time, 17.6%	18.3%	2%	41%
Szarewski [25]	UK	25-65 yrs; ≥6 years overdue	HPV SS kit	Standard invitation	Cotton swab	HCII, 8.3%	10.2%	4.5%	87.5%
Wikstrom [22]	Sweden	39-60 yrs; ≥6 years overdue	HPV SS kit* + 2^nd^ reminder	Standard invitation	Qvintip	HCII, 6%	39%	9%	98%
Rossi [26]	Italy	35-65 yrs; 3–5 months overdue	HPV SS kit	2 control arms	Pantarhei device	HCII , 1 = 21.8%, 4 = 6.5%	1 = 19.6%, 2 = 8.7%	3 = 13.9%, 4 = 14.9%	1 = 91%
			-Direct mail (1)*	-Standard recall (3)					
			-On demand (2)	-HPV at the clinic (4)					
Virtanen [23]	Finland	30-60 yrs; did not respond to primary invitation	HPV SS kit	Reminder letter	Delphi Screener	HCII	29.8%	26.2%	-
Virtanen [24]		30-60 years; did not respond to primary invitation	HPV SS kit*	Reminder letter	Delphi Screener	HCII, 12.3%	32%	26%	86.6%
Gok [27]	Netherlands	30-60 yrs; did not respond to invitation or 6 month reminder	HPV SS kit*	Second reminder letter*	Delphi screener	HCII, 10.3%	26.6%	16.4%	90.4%
Bais [28]		30-50 yrs, did not respond to invitation or 6 month reminder	HPV SS kit	Second reminder letter	Viba-brush + collection tube	GP 5+/6+ PCR, 8%	34.2%	17.6%	86%

*Pre-invitation letter informing of the arrival of the kit or reminder, and in Finland trial, this letter included “opt out” option.

All trials found participation to be significantly higher for HPV self-sampling than for a reminder to attend for Pap testing. However, the actual participation proportion in both the intervention (range 10% to 39%) and the control arms (range 2% to 26%) varied widely, with an absolute difference in participation between trial arms ranging between 3% and 30%. The studies also found a high compliance with follow-up regimens: between 86% and 98% of women who tested positive to HPV DNA underwent appropriate follow-up investigations [22,24-28] except for the trial in France [29]. There are no published trials that have had sufficient power to evaluate participation of never-screened women separately to under-screened. In summary, the trials show that mailing HPV self-sampling kits to non-attendees increases participation compared with standard reminder letters and that a high proportion of women who test positive undergo appropriate follow-up investigations. Because the participation fractions varied widely across countries, locally conducted trials are necessary to estimate the likely effect and cost effectiveness for a given country. Thus, we are conducting a randomised controlled trial to evaluate whether mailing an HPV self-sampling kit will increase participation in cervical screening in Victoria, Australia, when compared with a reminder letter to attend for a Pap test.

### Primary objective

To determine whether offering home-based HPV self-sampling increases participation in cervical screening, overall and separately for never- and under-screened women when compared to current practice of a reminder letter to attend for a Pap test.

### Secondary objectives

The main secondary objective is to estimate the proportion of women who have a positive HPV test who undergo appropriate further investigation, separately for never- and under-screened women. Other secondary objectives include documenting women’s experience with home-based HPV self-sampling, their willingness to participate in HPV self-sampling screening in future, and exploring reasons for non-participation.

## Methods

### Trial design

**Figure 1:**
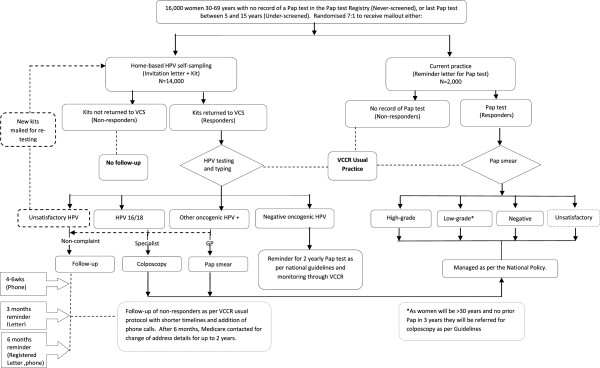
RCT design overview and clinical management for both never- and under-screened women.

We will conduct a parallel, randomized controlled, open label trial. Women will be randomly allocated to either the HPV self-sampling arm and receive an invitation letter with a kit for home-based self-sampling or the current practice arm of a letter prompting them to attend a health practitioner (General Practitioner (GP) or nurse) to have a Pap test (Figure 1). Women will be stratified by their screening history i.e. never- or under-screened.

To determine which women have no history of a previous Pap smear on the Victorian Cervical Cytology Registry (VCCR) and are thus presumably never-screened, we will link the VCCR to the enrolment information on the Victorian Electoral Commission (VEC). Registration to vote is compulsory in Australia and use of the electoral roll for specific public health programs, such as cancer screening, is permitted under legislation [30]. Women whose last recorded Pap test was between five and fifteen years ago will be defined as under-screened and identified using the VCCR database only. Under-screened women will be further stratified by years since last Pap test (i.e. 5 years, 6 years, 7 years, 8 years, 9 years, and 10–14 years), with equal numbers in each stratum. Women with a screening history whose last contact with VCCR was 15 years ago or more will probably be difficult to contact given their likelihood of having changed address. In previous VCCR studies of reminder letters, 35% of letters to women whose last Pap test was 15 years ago were “returned to sender” [11]. Within each stratum, women will be randomly allocated in a 7:1 randomization ratio to the intervention (HPV self-sampling) arm and the current practice arm respectively. The unequal allocation ratio is to ensure there is an adequate sample size in the HPV self-sampling arm to estimate precisely the proportion of women who have a positive HPV test who undergo appropriate further investigation.

### Study setting

The trial is based at the Victorian Cytology Service Inc (VCS). VCS hosts the VCCR, which records almost all Pap tests in Victoria (that is all tests except when a woman chooses not to have her records recorded on the register), and the VCS Pathology, which is a NATA (National Association of Testing Authorities, Australia) accredited laboratory that reports about half of those Pap test as well as HPV tests.

In Australia, the National Cervical Screening Program (NCSP) currently recommends that all women aged 18–69 years, who have ever been sexually active (regardless of HPV vaccination status), should have Pap smears every two years if they have no symptoms or history suggestive of cervical pathology [31]. The NCSP is supported by eight jurisdictional Pap test registers, of which VCCR is the Victorian operation. Like other registers, VCCR functions by: sending reminders to women when their Pap test is overdue, following up women with abnormal Pap test results where necessary, providing laboratories with screening histories to help with accurate reporting of tests, and providing quality assurance data to ensure the quality of reporting by laboratories. Cervical cytology and HPV results are sent to VCCR directly from reporting laboratories, as permitted by Victorian legislation. Almost all results are reported to the VCCR within one week, with longer delays for reporting histology, although most of the latter is reported within 3 months. The trial will make use of these existing infrastructures, including the VCCR follow-up processes and database (known as the Cytology information System (CIS)), which is designed to capture the relevant outcomes quickly and efficiently.

### Eligibility

#### Inclusion criteria

Participants will be Victorian residents, age 30–69 years, for whom there is either no record on the VCCR of a Pap test (never-screened) or the last recorded Pap test in the VCCR was between five and fifteen years ago (under-screened). Eligibility will be restricted to women 30 years of age or older given the low specificity of HPV DNA tests in younger women [17,32].

#### Exclusion criteria

**Figure 2:**
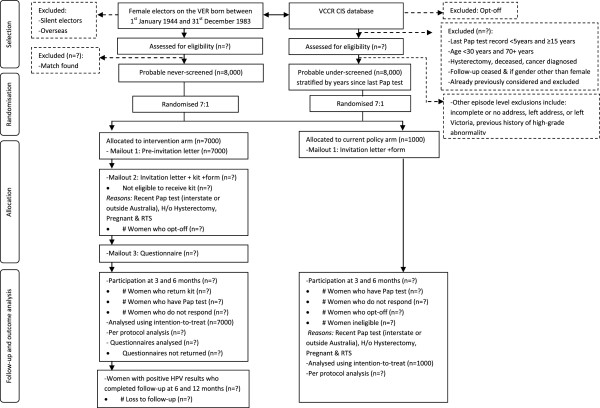
Flow of participants and timeline for women in the trial.

Different exclusion criteria apply to women in the two screening groups and at different stages of the participant selection process (Figure 2). For under-screened women, information on exclusion criteria is available prior to randomisation. Exclusions include women whose registry based follow up has ceased due to reported hysterectomy, gynaecological cancer, or migration, or those whose most recent Pap test showed a high-grade abnormality (these women require a different follow up pathway). Apart from age, no information on exclusion criteria for never-screened women is available prior to randomisation. Based on information reported by women following randomisation and mail-out, women will be deemed ineligible subsequently if found to be pregnant, if they have had a hysterectomy, if they have been recently screened (i.e. while interstate or overseas), or if the mail is returned.

### Interventions

Women allocated to the intervention (the HPV self-sampling) arm will be mailed an envelope containing an invitation letter, an information brochure on HPV and cervical cancer entitled *‘The Pap test alternative: the HPV test and cervical cancer’*, and the HPV self-sampling kit. The kit comprises a nylon-tipped flocked swab (Copan Italia, Brescia, Italy) for vaginal sampling enclosed in a plastic tube within a resealable plastic bag; an instruction sheet (both written and pictorial) on ‘*How to take a vaginal sample and how to pack and post the sample’*; a pathology information form; and a postage paid envelope to return the swab and the form. The form will ask for the woman’s country of birth, language spoken at home, whether she identifies as an Aboriginal or Torres Strait Islander woman, hysterectomy status, pregnancy status, Pap screening history, updates of her contact details, and the date she took her sample. Women are able to nominate a GP to receive a copy of the results to enable appropriate referral, follow-up and management should high-risk HPV be detected.

Two to three weeks prior to receiving the kit, women will receive a pre-invitation letter informing them of the upcoming HPV self-sampling kit and the fact that the test is free, and a phone number for calling the Registry (or VCCR) with an option for cancellation of the kit. It will also allow women to call the Registry and update information such as a recent Pap test, correct contact details, hysterectomy, or pregnancy to identify women not eligible for the trial. A multilingual flyer included with the pre-invitation and the invitation letter will state that information is available on the website in the ten most common languages and that an interpreter service is available.

All materials for use in the HPV self-sampling arm were focus group tested. Four focus groups were conducted in August 2013 with Victorian women who would be eligible for the trial, separated by their screening history (never- and under-screened) and age (30–49 and 50–69 years). The main aim was to obtain suggestions for refining all written materials sent with the HPV self-sampling kit with the intent of maximising response to the trial. The overall response to the iPap concept was very positive in the focus groups. The details of the focus group findings will be published elsewhere.

Women in the HPV self-sampling arm will also receive a questionnaire a few weeks later. This will collect information about their experience with the HPV self-sampling (mostly psycho-social: pain, discomfort, fear, embarrassment; some aspects of feasibility such as ease of use, confidence doing it themselves, adequacy of instructions; and other practical issues such as ease getting an appointment with a GP etc.) and their willingness to participate in HPV self-sampling screening in future. Those who did not return a completed HPV self-sampling kit will be asked to provide reasons for not participating.

Women allocated to the current practice arm will receive either a tailored invitation letter (never-screened) or a standard reminder letter (under-screened) to have a Pap test, as well as a Pap test brochure entitled *‘How Pap tests can help prevent cervical cancer, and the Pap test registry’.* Also included will be a form to collect the similar information as for the HPV self-sampling arm, and a reply paid envelope for return of the form. The requested information will enable analysis of results by cultural background and Indigenous status, and also identify women not currently eligible for screening.

### Laboratory testing of HPV

All the kits received will be handled, processed and tested by VCS Pathology using the Cobas® 4800 HPV Test (Roche Diagnostics GmBH) according to the manufacturer’s instructions. The test is clinically validated and approved by the FDA. The test specifically identifies high-risk types HPV16 and HPV18 while concurrently detecting 12 other high-risk types (31, 33, 35, 39, 45, 51, 52, 56, 58, 59, 66 and 68) in a single pool at clinically relevant cut offs for detecting infection. This test allows for risk stratification and identification of women who are at the highest risk of cervical cancer (those who are 16/18 positive) [33] who may need more intensive follow-up. The Cobas® HPV test also has high sensitivity, which is desirable for under-screened women [34,35]; a Beta-globin internal control for sample adequacy, which will reduce false-negatives; and a lower rate of cross-reactivity with low-risk HPV types, reducing false-positives [36]. Results are either ‘positive’ for high-risk HPV (HPV 16, HPV 18 and/or other high-risk HPV types) or ‘negative’ for high-risk HPV or unsatisfactory. An ‘unsatisfactory’ result includes specimens damaged in transit; incorrect labelling; non return of the pathology form; inhibition by blood or other substance; or insufficient material to test.

### Clinical management

Women will be sent a letter notifying them of their HPV result directly, with a copy to their nominated GP (if provided) within two weeks of testing. GP correspondence will detail the study, HPV result and recommended follow-up and management. The letter to the women will be accompanied by appropriate educational information on the meaning of the test result and the recommended follow-up or clinical management. Figure 1 shows the proposed clinical management for women in the trial. Women in the intervention arm who test negative for high-risk HPV will be informed of the result and advised to have regular Pap tests as per the current screening policy. Women positive for high-risk types other than HPV16 or HPV18 will be asked to visit their GP for a Pap test. Cytology will be reported as per the Australian Modified Bethesda System [37]. Women with abnormal Pap test results (≥ possible low-grade squamous intraepithelial lesion) will be referred for colposcopy by their GP to a specialist of their choice. Women whose samples test positive for high-risk HPV16 and/or HPV18 will be directly referred for colposcopy. Colposcopy directed biopsies will be taken for histological examination from the cervix if clinically indicated and managed as per National Health and Medical Research Council (NHMRC) guidelines [37]. Histological results will be sent to the VCCR as per usual practice, where they are coded in a standard manner and undergo quality assurance checking. Women with positive high-risk HPV but negative colposcopy or cytology will be followed up actively through their GP and advised to undergo screening (either a repeat HPV test and/or Pap) after a year. As future screening will be outside the timeline of this study, we cannot offer a further HPV self-sample to these women and hence further screening will be as is recommended by the NCSP, which is presently 2 yearly Pap testing (this is currently under review) [38]. In case of an unsatisfactory result, new kits will be mailed to women.

Women with high-risk HPV positive results but without a GP will be strongly recommended to contact a GP or arrange referral to a gynaecologist for further follow-up. In the case of HPV 16 and/or HPV18 positive results, women will be contacted by the VCS Liaison Physician to help them seek further medical advice. Follow-up of non-compliant women with positive results will be as per VCCR usual protocol for follow up of high grade cytology with shorter timelines and addition of phone calls. After 6 months, Medicare (Australia’s publicly funded universal health care system) will be contacted to supply any change of address details held for the woman for up to 2 years. There will be no follow-up of women who chose not to respond to the kit or the reminder letter, with the exception of a participation questionnaire sent to intervention non-responders. Women in the current practice arm with abnormal Pap test results will be managed as per NHMRC guidelines by their treating doctor or nurse. Because the women who participate in either trial arm will be part of the screening program, in other words their test results will be recorded on the VCCR; they will receive future reminders from VCCR at the appropriate time interval depending on their screening result.

### Outcomes

The primary outcome is the return of a completed HPV self-sampling kit or the notification of a Pap test result to the VCCR per trial arm. Women in the HPV self-sampling arm who attend for a Pap smear instead of self-sampling will be counted as ‘successes’. Outcomes will be measured at 3 and 6 months after the mailout of the kits or letters. The secondary outcome is whether women who have a positive high-risk HPV test undergo appropriate further investigation i.e. either have a Pap test or colposcopy depending on the type of high-risk HPV detected. This will be measured 6 and 12 months after women are informed of their results.

### Participant timeline

The expected study duration is 36 months. Mailout of kits and letters will occur progressively in batches over about 4 months beginning March 2014. Results will be mailed out within two weeks of testing and follow-up monitored in the next 6–12 months of the mailout of the test positive letter. The questionnaire will be mailed to women, with differing timelines for responders and non-responders.

### Sample size

The sample size was determined by the secondary objective (the proportion of women who have a positive HPV test who undergo appropriate further investigation). We will contact 16,000 women; 8,000 women never-screened and 8,000 women under-screened. 7,000 women will receive the HPV self-sampling kit within each screening group. Based on an estimate of 20% participation and prevalence of positive result for HPV test of 10%, there will be at least 140 women invited for a follow-up. With 140 women we will obtain a 95% confidence interval of +/− 5% points around an estimated follow-up proportion of 90%. Table 2 shows estimated power for different participation fractions in the two arms of the trial (assuming 1000 women in the comparison arm (n1) and 7000 in the HPV self-sampling arm (n2)). Our assumption of the participation fraction in the current practice arm (i.e. women who will have a Pap test within 3 months of receiving the reminder letter) is based on a pilot study of second reminder letters, where the response fractions varied between 0.4%, 2% and 10% for women whose time since last Pap test was fifteen, ten and six years respectively [11]. The sample size adequately accounts for ineligible women (e.g. those pregnant; history of hysterectomy; or those recently screened and not recorded in the Registry) and the return to sender received during mailout. We assumed a 30% hysterectomy rate, 10% pregnancy rate and a 30% return to senders in addition to 20% participation and 10% non-responders. The hysterectomy fraction is a conservative estimate as data from the National Hospital Morbidity identifies rates for 30–69 years range from 2% (in 30–34 years) to 30% (in 65–69 years) [2]. The return to sender rates estimates are likewise conservative as figures from the second reminder pilot study are 19% (in women whose last Pap test was 5–9 years ago), 27% (in women whose last Pap test was 10 to 14 years ago) and 35% (in women whose last Pap test was 15 years ago) [11].

**Table 2:** Power calculations assuming different participation fraction in the two arms of the trial

**Participation**		**Statistical power for primary aim**
**Comparison**	**HPV self sampling**	
**(n1 = 1000)**	**(n2 = 7000)**	
2%	7%	100%
2%	10%	100%
5%	8%	94%
10%	14%	95%
15%	19%	87%
5%	7.4%	80%

### Selection of participants

The electoral roll from the VEC will constitute the sampling frame for never-screened women. The electoral roll will include female Victorian electors born between 1 January 1944 and 31 December 1983, excluding silent and overseas electors. A subset of women will be selected by simple random sampling (using a computer generated random number) and a check will be made against the VCCR CIS database to find a match. Women will be matched on name, address and date of birth and if no match is found, they are eligible for inclusion as never-screened women. If insufficient eligible women are found in the first random subset, another will be selected and the process repeated.

Under-screened women will be identified directly from the VCCR CIS database after excluding women whose last Pap test was <5 years and ≥15 years ago, age <30 years or 70+ years, who have a prior history of cancer, hysterectomy, are deceased, or whose follow-up has ceased. A subset of women will then be selected by simple random sampling (using a computer generated random number) and further considered for: completeness of mailing address, or a change of address, most recent Pap test showing no high-grade abnormality and having not recently migrated or moved. The two-stage process is necessary because the second stage requires assessing episode level information. At this point, if all the above criteria are met, selected under-screened women are considered eligible. Under-screened women will then be grouped into strata (5 years, 6 years, 7 years, 8 years, 9 years, and 10–14 years since last Pap) with approximately equal numbers in each stratum.

### Randomisation

Selected women will be randomly allocated to the two arms of the trial in a 7:1 (HPV self-sampling: current practice) ratio as per a computer generated randomisation schedule stratified by screening history and time since last Pap test (for under-screened only) within blocks or batches of fixed size. The batches will ensure a close balance of numbers in each arm at any time during the trial and will enable us to regulate the administrative work flow as well as the work flow in the laboratory. This will also enable us to monitor participation and contain expenditure if the response is higher than anticipated.

The study ID and no other information will be used for the randomisation. The nature of the intervention and the randomization ratio precludes masking of participants and other study staff, including the data analyst.

### Data plan

#### Data collection

There are a number of ways in which participant information will be returned and collected. Women can post back their pathology information form, call the telephone centre and update details, or return the completed kit (with the form), or staff at VCCR process the return to sender generated during mailout. The data flow will go through several checkpoints of information matching and validation prior to entry, amendment of details or sample processing. Additionally, women in the self-sampling arm will return the questionnaire by post. These questionnaires will be anonymous, therefore when they are returned they cannot be linked to a specific woman.

#### Data analysis

Within each stratum (never- and under-screened) the proportion of women in each arm participating (i.e. primary outcome) will be calculated as will the absolute difference in the proportion of participation between the HPV self-sampling and current practice arms and the corresponding 95% confidence interval and two-sided p-value. An intention-to-treat approach will be used for the analysis, with women who subsequently report they are adequately screened, are pregnant, or have had a hysterectomy, or return to sender, analysed within the arm to which they were randomised. We will also calculate adjusted participation proportions, in which women who report that they are pregnant, have had a hysterectomy, or that they are adequately screened, or return to sender, are removed from the denominators. Participation will be also be reported by age, socio-economic status (SES), cultural background, Indigenous status and time since last Pap test (for under-screened only) within each screening group and trial arm status. SES status classification is an area level variable assigned to women according to their postcode of residence based on the ABS Index of Relative Socioeconomic Disadvantage (ABS 2008) which is derived from Census information. Additionally, the distribution of age and SES will be compared between women who participate and those who don’t within each screening group and trial arm. Estimates and 95% confidence intervals of the proportion of women having appropriate further investigation (i.e. secondary outcome) in the overall and sub-groups of positive HPV test for each screening group will also be calculated. The responses to the different items (i.e. items within the themes: psycho-social, feasibility and practical issues) of the self-sampling follow-up questionnaire will be summarised and reported as the total number and proportion. We will also report the reasons for not returning a self-sampling kit.

#### Data security

VCCR has strict data security arrangements, data privacy and data linkage policies and protocols in place to ensure the privacy and confidentiality of the personal information that it holds. Additional conditions surround the VEC data which include all staff involved signing confidentiality agreements, to ensure that the information remains confidential and protected. The information provided by the VEC has been allowed based on the public health benefit of this research and will not be used for any other purposes. The collating of the letters and/or letters and kits and inserting into envelopes is performed in a specifically designed and purpose built area with limited staff access.

In accordance with VEC requirements, VCCR will fully destroy the information collected from VEC at three months from the date of release of the electoral roll from VEC, except for women selected for the study. The details of women selected for the study who do not participate will be deleted after 12 months. Year of birth and postcode will however be retained for statistical purposes. Notably when a never-screened woman returns the kit and consents to her information being recorded on the VCCR this information is no longer VEC data and becomes VCCR data. The final data set for analysis will be de-identified and published in an aggregate manner so confidentiality will be protected.

### Ethics

The study was approved by the Human Research Ethics Committee (HREC) of the Victorian Department of Health. Informed consent has been waived for the study because it is primarily a trial of participation in screening, and we wish the results to be directly translatable to the current screening program. Asking women to consent to a trial of participation would potentially invalidate such results. However, women are otherwise fully informed about the testing and subsequent follow-up and once a woman returns the kit with a pathology information form it will represent her consent to test her sample and undergo further appropriate management and use of data. Correspondence sent to women includes a clear statement indicating the process under which VCCR obtained addresses from the electoral roll and participant information form includes statements about privacy of information. Additional information on how the data collected will be stored and utilized in the study is detailed in the brochure sent with the kit or the reminder letter. Provision has been offered for women to opt-off the study. VCCR will inform VEC of women who opt-off and who do not want to be contacted for health screening purposes in future. All materials were revised following focus groups and amended materials submitted for consideration by HREC.

A Data Safety Monitoring Board (DSMB) has been appointed for the duration of the study. The DSMB will be responsible for the review of the primary outcome data. The DSMB will be provided with the full outcome data at six and twelve months post mail-out of the invitation letter. Following review of the data, the DSMB will report back to the Principal investigator with written details of any identified issues and/or recommendations. An interim analysis will also be performed on the primary end point when 50% of the mail-outs have occurred (anticipated to take place around the 3^rd^ month post initial contact). The DSMB could halt or extend the study based on the primary end points. They will also provide clinical advice on detected high-risk HPV where a woman is non-compliant with follow-up, and assess VCCR compliance of follow-up reminders based on participants becoming part of the routine screening program.

## Discussion

The success of the National Cervical Screening Program is limited by incomplete and unrepresentative participation. More than half of invasive cervical cancers presently occur in women who have never been screened and close to a quarter occur in women who are under-screened. Improving participation and reducing inequalities are important priorities for the cervical screening program and new strategies are needed to target the hardest to reach women. HPV self-sampling is a valid screening test that performs better than cervical cytology in detecting abnormalities and has the potential to overcome known barriers to screening, as trials have shown it can improve participation by hard-to-reach groups, although the participation rates varied widely between countries. Given the importance of the local context to screening participation, evidence from Australian trials is necessary to inform policy. None of the previous published trials have had sufficient power to evaluate participation of never-screened women separate to under-screened. This novel aspect of the study has great public health significance as these women are hardest to reach and have the highest rate of cervical cancer. A pragmatic trial of HPV self-sampling is timely and the findings will have direct relevance to the cervical screening program.

## Competing interests

MS is the Principal investigator of the COMPASS trial of primary HPV screening in Australia that has received funding contribution from Roche Molecular Systems USA. DG, SH, DW and JB are co-investigators on the COMPASS trial. No funding from Roche has been received for the purpose of the iPap trial. All other authors declare no other conflicts of interest.

## Authors’ contributions

DG is the principal investigator of the study and is responsible for the overall conduct of the study. FS developed the first draft of the protocol that was further developed by DG, DE, FS, JAS, JB and MS. DG, DE, MS, JB, JAS and FS are primarily responsible for the design of the study, with input from all authors. FS is a PhD student doing her PhD on the topic and will be responsible for scientific coordination of the trial, statistical analysis and manuscript preparation, with oversight from DE, DG, JAS, JB and MS. KD is managing the operational coordination of the study. MS will oversee the laboratory testing for self-sampled HPV, reporting of results and ensure laboratory quality assurance. DW and SH will provide clinical advice on follow-up of women with positive results. RM was responsible for oversight of the focus groups. All authors read and approved the final manuscript.

## Pre-publication history

The pre-publication history for this paper can be accessed here:

http://www.biomedcentral.com/1471-2407/14/207/prepub
